# Supplementary material for: Increasing Black, Indigenous and People of Color participation in clinical trials through community engagement and recruitment goal establishment
Source: PLoS One. 2021 Oct 19;16(10):e0258858. doi: 10.1371/journal.pone.0258858 (PMC8525736; doi:10.1371/journal.pone.0258858)

PLOS ONE

Increasing Black, Indigenous and People of Color Participation in Clinical Trials Through Community Engagement and Recruitment Goal Establishment

--Manuscript Draft--

**Manuscript Number:**	PONE-D-21-14138
**Article Type:**	Research Article
**Full Title:**	Increasing Black, Indigenous and People of Color Participation in Clinical Trials Through Community Engagement and Recruitment Goal Establishment
**Short Title:**	Increasing Black, Indigenous and People of Color Participation in Clinical Trials
**Corresponding Author:**	Michele Peake Peake, PhD, EdM<br>Fred Hutchinson Cancer Research Center<br>Seattle, WA UNITED STATES
**Keywords:**	HIV; COVID-19; Vaccines; Clinical Trials; Ethnic & Racial Inclusion; Equity
**Abstract:**	Longstanding social and economic inequities elevate health risks and vulnerabilities for Black, Indigenous and People of Color (BIPOC) communities. Engagement of BIPOC communities in infectious disease research is a critical component in efforts to increase vaccine confidence, acceptability, and uptake of future approved products. Recent data highlight the relative absence of BIPOC communities in vaccine clinical trials. Intentional and effective community engagement methods are needed to improve BIPOC inclusion. We describe the methods utilized for the successful enrollment of BIPOC participants in the NIH-funded COVID-19 vaccine efficacy trials and analyze the demographic and enrollment data across the efficacy trials to inform future efforts to ensure inclusive participation. Across the four US government-funded COVID-19 vaccine clinical trials for which data are available, 47% of participants enrolled at CoVPN sites in the US were BIPOC. White enrollment outpaced enrollment of BIPOC participants throughout the accrual period, requiring the implementation of strategies to increase diverse and inclusive enrollment. Trials opening later benefitted considerably from ongoing community engagement efforts, and greater and more diverse volunteer registry records. With ample resources and community engagement expertise, the equitable enrollment of BIPOC individuals can be achieved. Despite robust fiscal resources and a longstanding collaborative and collective effort, enrollment of White persons outpaces that of BIPOC communities. To ensure the equitable inclusion of BIPOC communities, intentional efforts are needed. These include an emphasis on diversity of enrollment in clinical trials, establishment of enrollment goals, ongoing robust community engagement, and conducting population-specific trials.
**Order of Authors:**	Michele Peake Andrasik, PhD, EdM
	Gail B. Broder
	Stephaun E. Wallace
	Richa Chaturvedi
	Nelson L. Michael
	Sally Bock
	Chris Beyrer
	Linda Oseso
	Jasmin Aina
	Jonathan Lucas
	David R. Wilson
	James Kublin
	George A. Mensah
**Additional Information:**	

Powered by Editorial Manager® and ProduXion Manager® from Aries Systems Corporation

Question	Response
**Financial Disclosure**<br><br>Enter a financial disclosure statement that describes the sources of funding for the work included in this submission. Review the submission guidelines for detailed requirements. View published research articles from *PLOS ONE* for specific examples.<br><br>This statement is required for submission and **will appear in the published article** if the submission is accepted. Please make sure it is accurate.<br><br>**Unfunded studies**<br>Enter: *The author(s) received no specific funding for this work.*<br><br>**Funded studies**<br>Enter a statement with the following details:<br>• Initials of the authors who received each award<br>• Grant numbers awarded to each author<br>• The full name of each funder<br>• URL of each funder website<br>• Did the sponsors or funders play any role in the study design, data collection and analysis, decision to publish, or preparation of the manuscript?<br>• **NO** - Include this sentence at the end of your statement: *The funders had no role in study design, data collection and analysis, decision to publish, or preparation of the manuscript.*<br>• **YES** - Specify the role(s) played.<br><br>* typeset	All authors were funded by the NIH Institutes of Health through the COVID-19 Prevention Network. Dr. Chris Beyrer received support from the CoVPN for his community engagement work.
**Competing Interests**<br><br>Use the instructions below to enter a competing interest statement for this submission. On behalf of all authors, disclose any competing interests that could be perceived to bias this work—acknowledging all financial support and any other relevant financial or non-financial competing interests.	The authors have declared that no competing interests exist.

This statement is required for submission and **will appear in the published article** if the submission is accepted. Please make sure it is accurate and that any funding sources listed in your Funding Information later in the submission form are also declared in your Financial Disclosure statement.<br><br>View published research articles from *PLOS ONE* for specific examples.<br><br>**NO authors have competing interests**<br><br>Enter: *The authors have declared that no competing interests exist.*<br><br>**Authors with competing interests**<br><br>Enter competing interest details beginning with this statement:<br><br>*I have read the journal's policy and the authors of this manuscript have the following competing interests: [insert competing interests here]*<br><br>* typeset	
**Ethics Statement**<br><br>Enter an ethics statement for this submission. This statement is required if the study involved:<br><br>• Human participants<br>• Human specimens or tissue<br>• Vertebrate animals or cephalopods<br>• Vertebrate embryos or tissues<br>• Field research<br><br>Write "N/A" if the submission does not require an ethics statement.<br><br>General guidance is provided below. Consult the submission guidelines for detailed instructions. **Make sure that all information entered here is included in the Methods section of the manuscript.**	Across all COVID-19 protocols, IRB approval was obtained at the respective institutions for the local clinical research sites

**Format for specific study types**<br><br>**Human Subject Research (involving human participants and/or tissue)**<br>• Give the name of the institutional review board or ethics committee that approved the study<br>• Include the approval number and/or a statement indicating approval of this research<br>• Indicate the form of consent obtained (written/oral) or the reason that consent was not obtained (e.g. the data were analyzed anonymously)<br><br>**Animal Research (involving vertebrate animals, embryos or tissues)**<br>• Provide the name of the Institutional Animal Care and Use Committee (IACUC) or other relevant ethics board that reviewed the study protocol, and indicate whether they approved this research or granted a formal waiver of ethical approval<br>• Include an approval number if one was obtained<br>• If the study involved *non-human primates*, add *additional details* about animal welfare and steps taken to ameliorate suffering<br>• If anesthesia, euthanasia, or any kind of animal sacrifice is part of the study, include briefly which substances and/or methods were applied<br><br>**Field Research**<br><br>Include the following details if this study involves the collection of plant, animal, or other materials from a natural setting:<br>• Field permit number<br>• Name of the institution or relevant body that granted permission	
**Data Availability**<br><br>Authors are required to make all data underlying the findings described fully available, without restriction, and from the time of publication. PLOS allows rare exceptions to address legal and ethical concerns. See the PLOS Data Policy and FAQ for detailed information.	Yes - all data are fully available without restriction

Powered by Editorial Manager® and ProduXion Manager® from Aries Systems Corporation

A Data Availability Statement describing where the data can be found is required at submission. Your answers to this question constitute the Data Availability Statement and **will be published in the article**, if accepted.<br><br>**Important:** Stating 'data available on request from the author' is not sufficient. If your data are only available upon request, select 'No' for the first question and explain your exceptional situation in the text box.<br><br>Do the authors confirm that all data underlying the findings described in their manuscript are fully available without restriction?	
**Describe where the data may be found in full sentences. If you are copying our sample text, replace any instances of XXX with the appropriate details.**<br><br>• If the data are **held or will be held in a public repository**, include URLs, accession numbers or DOIs. If this information will only be available after acceptance, indicate this by ticking the box below. For example: *All XXX files are available from the XXX database (accession number(s) XXX, XXX.*).<br>• If the data are all contained **within the manuscript and/or Supporting Information files**, enter the following: *All relevant data are within the manuscript and its Supporting Information files.*<br>• If neither of these applies but you are able to provide **details of access elsewhere**, with or without limitations, please do so. For example:<br><br>*Data cannot be shared publicly because of [XXX]. Data are available from the XXX Institutional Data Access / Ethics Committee (contact via XXX) for researchers who meet the criteria for access to confidential data.*<br><br>*The data underlying the results presented in the study are available*	All relevant data are within the manuscript and its Supporting Information files

from (include the name of the third party and contact information or URL).<br>• This text is appropriate if the data are owned by a third party and authors do not have permission to share the data.<br><br>* typeset	
Additional data availability information:	Tick here if the URLs/accession numbers/DOIs will be available only after acceptance of the manuscript for publication so that we can ensure their inclusion before publication.

Powered by Editorial Manager® and ProduXion Manager® from Aries Systems Corporation

Manuscript

Click here to access/download;Manuscript;Andrasik_RobustCESettingGoals_

Increasing Black, Indigenous and People of Color participation in clinical trials through community engagement and recruitment goal establishment

Michele P. Andrasik[1#+], Gail B. Broder[1#], Stephaun E. Wallace[1#], Richa Chaturvedi[2#], Nelson L. Michael, MD[3#], Sally Bock[4*], Chris Beyrer[5*], Linda Oseso[1*], Jasmin Aina[1*], Jonathan Lucas[6*,] David R. Wilson[7*], James Kublin[1*], George A. Mensah[8#]

[1]Vaccine and Infectious Disease Division, Fred Hutchinson Cancer Research Center, Seattle, WA

[2]Statistical Center for HIV/AIDS Research and Prevention, Fred Hutchinson Cancer Research Center, Seattle, WA

[3]Walter Reed Army Institute of Research, Silver Spring, MD

[4]Fred Hutchinson Cancer Research Center, Seattle, WA

[5]John's Hopkins Bloomberg School of Public Health, Baltimore, MD

[6]HIV Prevention Trials Network, FHI360, Research Triangle, NC

[7]Tribal Health Research Office, National Institutes of Health, Bethesda, MD

[8]National Heart, Lung, and Blood Institute, National Institutes of Health, Bethesda, MD

+ Corresponding Author

Email: mandrasik@fredhutch.org (MA)

#These authors contributed equally to this work.

*These authors also contributed equally to this work.

Abstract:

Longstanding social and economic inequities elevate health risks and vulnerabilities for Black, Indigenous and People of Color (BIPOC) communities. Engagement of BIPOC communities in infectious disease research is a critical component in efforts to increase vaccine confidence, acceptability, and uptake of future approved products. Recent data highlight the relative absence of BIPOC communities in vaccine clinical trials. Intentional and effective community engagement methods are needed to improve BIPOC inclusion. We describe the methods utilized for the successful enrollment of BIPOC participants in the NIH-funded COVID-19 vaccine efficacy trials and analyze the demographic and enrollment data across the efficacy trials to inform future efforts to ensure inclusive participation. Across the four US government-funded COVID-19 vaccine clinical trials for which data are available, 47% of participants enrolled at CoVPN sites in the US were BIPOC. White enrollment outpaced enrollment of BIPOC participants throughout the accrual period, requiring the implementation of strategies to increase diverse and inclusive enrollment. Trials opening later benefitted considerably from ongoing community engagement efforts, and greater and more diverse volunteer registry records. With ample resources and community engagement expertise, the equitable enrollment of BIPOC individuals can be achieved. Despite robust fiscal resources and a longstanding collaborative and collective effort, enrollment of White persons outpaces that of BIPOC communities. To ensure the equitable inclusion of BIPOC communities, intentional efforts are needed. These include an emphasis on diversity of enrollment in clinical trials, establishment of enrollment goals, ongoing robust community engagement, and conducting population-specific trials.

Introduction:

The persistent and pervasive health inequities experienced by Black, Indigenous and People of Color (BIPOC) communities are well documented[1,2]. Longstanding structural inequities elevate health risks and vulnerabilities[3]. When faced with infectious diseases, disparities in morbidity and mortality rapidly emerge in BIPOC communities. Effectively engaging BIPOC communities in clinical research is critical to addressing the history of research ethical abuses and the development of trustworthy reputations and relationships. ~~Setting~~ these conditions should increase vaccine confidence, acceptability, and uptake when approved products become available, thereby strengthening public health.

When the HIV Vaccine Trials Network (HVTN) Leadership Operations Center became part of the COVID-19 Prevention Network (CoVPN) in March 2020, the Community Engagement Team led community engagement efforts for the US government-funded COVID-19 vaccine efficacy trials. Longstanding HIV community engagement efforts enabled a quick pivot to address COVID-19.

A robust community engagement effort necessitates relationship building, trustworthiness, and bidirectional communication. The HVTN has worked to center community engagement across its preventive HIV vaccine trial efforts since its founding in 1999.

Community members are at the heart of these efforts; and without community, moving impactful science forward is impossible. Central to these efforts is the utilization of Good Participatory Practice[4] as a framework, and behavioral theories[5-7] to guide the work. Across the HVTN, particularly at clinical research sites (CRSs), community engagement is a collective responsibility shared by persons in all roles - investigators, community staff, clinicians, and Community Advisory Board (CAB) members - and across the entire lifecycle of a research endeavor. CAB members are also included on every HVTN protocol team, operational and scientific committee, and working group. Support for building community engagement capacity is available to CAB members to ensure meaningful engagement and contributions.

All CRSs are required to have active community advisory groups with clear lines of communication to CRS staff and leadership. CRSs are also required to develop annual work plans that outline processes and goals for community engagement efforts with measurable objectives that are reviewed and approved by the HVTN Community Engagement Team. Community Working Groups (CWGs) comprised primarily of community staff and CAB members are convened to provide guidance and direction for all efficacy trials.

Additionally, the HVTN conducts ongoing mixed methods studies to inform an increased understanding of barriers and facilitators to research participation for populations most impacted by HIV[8-14]. In 2017, we examined demographic characteristics across Phase 1 – 2a preventive HIV vaccine studies conducted in the US[15]. Prioritizing community partnerships and investing resources in community engagement showed a 94% increase in enrolled participants who identified as a member of a racial/ethnic minority group, increased from 17% in trials conducted between 1988 and 2002[16] to 32.7% in trials conducted between 2002 and 2016.

Recent data illustrate the need for effective efforts to ensure equitable inclusion of BIPOC communities in vaccine clinical trials[17]. BIPOC communities are disproportionately impacted by COVID-19 cases, hospitalizations, and deaths[18,19]. Ensuring BIPOC enrollment in the COVID-19 vaccine trials was critical to ensure that the vaccines were evaluated in the context of their intended use, and to support inclusive vaccine acceptance and uptake efforts. We describe the methods utilized for the successful enrollment of BIPOC participants in the US government-funded COVID-19 vaccine efficacy trials, and analyze the related demographic and enrollment data to inform future efforts on inclusive participation.

Materials and Methods:

Pivoting to COVID-19 work required reaching out to new and existing partners, engaging in conversations to understand how COVID-19 was impacting their respective communities, exploring barriers to trial participation and challenges to vaccine confidence and acceptability, and identifying processes to ensure community input into research protocols and community engagement efforts. These conversations informed a five-part CoVPN community engagement strategy that was executed by the CoVPN, supplementing local efforts undertaken by the CRSs.

Part I: Utilization of Community-Based Participatory Research approaches

Increasing community awareness and knowledge to address and correct misperceptions, misinformation, and myths required the utilization of Community-Based Participatory Research (CBPR) approaches[20] and working with partners such as the NIH

Community Engagement Alliance (CEAL) Against COVID-19 Disparities (https://covid19community.nih.gov/). These efforts included developing community engagement. Involving community members and leaders in this process from the beginning ensured the use of respectful language (e.g., older adult vs. elderly, priority vs. target populations, American Indian/Alaska Native vs. Native Americans), inclusive identifiers (e.g., Native AND Indigenous; Asian AND Pacific Islander) and comprehensible materials (e.g., explaining safety pauses; expedited vaccine development processes; prevention of severe disease vs. prevention of acquisition). Access to these materials and information in English and Spanish was facilitated through the development of a US-focused website, the use of a toll-free call center with Spanish language capacity, and a publicly accessible Dropbox (TinyURL.com/CoVPN-Assets **Password:** CoVPNTria!$).

In addition to general public education, the materials drove COVID-19 vaccine inquiries to the website by referencing the URL (www.PreventCOVID.org). Each page on the website included a prominently displayed "Volunteer Now" link~~, which~~ directed interested parties to the Volunteer Screening Registry and its pre-screening survey. The survey collected contact information, demographics, and risk factors relating to employment and living conditions as predictors for risk of SARS-CoV-2 acquisition and development of severe COVID-19 illness. All US CRSs conducting COVID-19 vaccine studies had access to the pool of volunteers living in a set of pre-determined local zip codes. The registry database supported the enrollment of 30,000 or more people for each of the four CoVPN COVID-19 vaccine efficacy trials [Moderna, AstraZeneca (AZ), Janssen, and Novavax] and allowed the CRSs to contact interested individuals about specific trials. Use of the Registry also allowed sites to focus their outreach efforts to particular demographics of interest as well as different risk factors as specified in any given clinical trial. As of April 2021, the registry has over 600,000 diverse individuals who completed the survey. Efforts are currently underway to expand the registry to include pediatric populations, gather data on SARS-CoV-2 infection, experiences with long-term COVID-19 disease, and to support future trials.

Part II: Involving communities throughout the research process

Effective community engagement involves community members at all stages of the research. As protocols were being developed, a CoVPN CWG ~~comprised~~ of CRS community engagement staff and CAB members was convened to offer insight into needed educational materials, review materials in development, and provide general guidance and direction for community engagement efforts. A CAB member and a Community Educator representative were also involved in reviewing each efficacy protocol and providing input into the informed consent materials. Ongoing capacity building and skills development ensured that community members had the tools and skills needed to meaningfully engage in protocol discussions.

In early conversations, our partners from the Indigenous Wellness Research Institute recommended the development and convening of priority population expert panels to discuss each protocol in development, and generate reports detailing considerations and actions needed to ensure inclusion of BIPOC and older adult communities. Four US-based and one Latin American expert panel were convened (Native/Indigenous; Black/African American; Hispanic/Latino/a; Older Adult/Veterans). Each panel's members included 10-15 scientists and community leaders who identified with their respective priority population; represented

diverse biomedical, social, and behavioral science expertise; and dedicated their professional life to working with and within their communities.

Relationships established with these groups were vital in the success of our efforts to onboard CRSs at the four US Historically Black Medical Colleges and engage tribal and indigenous communities and their leaders, addressing tribal sovereignty and trial data ownership rights[21]. Ongoing communication with these panels highlighted the need to address social determinants of health[22] and informed the early initiation of efforts to reduce participation burdens and costs for participants through the establishment of incentives that adequately reimbursed participants for their travel and other related study costs, as well as the acquisition and utilization of mobile units and satellite clinics, taking research to communities.

Part III: Stakeholder engagement and building trust

Working in partnership with institutions and organizations with whom longstanding trusting relationships have been established is a vital component of community engagement, particularly in BIPOC communities who have a long history of and contemporary experiences with institutional racism and research ethics abuses. The HVTN and its CRSs have relied on such partnerships to assist in our efforts to communicate and share information with communities and potential participants with humility and authenticity. COVID-19 quickly became politicized, increasing perceptions of systems and research institutions as untrustworthy, and creating fear and uncertainty about the vaccine clinical trials and vaccination in general. Partnerships were instrumental in developing and implementing activities that utilized trusted voices to address questions and concerns about safety and side effects, equitable inclusion in vaccine trials, and the pace of vaccine development. These partnerships included social service providers, advocacy organizations, physician and medical professional associations, media, academic institutions, local/state/national government partners, and faith-based organizations, particularly those who serve BIPOC communities. Outreach to essential worker organizations and corporations, such as meat packing plants, nursing homes and assisted living facilities, and agricultural industries was critical to the COVID-19 efforts. Forming these new partnerships opened important channels of communication and information dissemination. Building and enhancing trust also involved utilizing our partnerships with leaders of these organizations as "trusted voices," persons to whom community members could ask questions and obtain the information needed to make informed health and medical decisions. Our efforts in this area also supported bringing many of these organizations and community leaders together to co-coordinate and co-host COVID-19 education sessions for their communities, providing another opportunity for communities to see unified trusted voices sharing science and addressing community concerns regarding COVID-19.

Part IV: A faith initiative

The CoVPN Faith Initiative leveraged the breadth of established relationships from the HVTN's history of successful engagement in faith communities. Through these efforts, a faith-based advisory council was established to provide guidance and direction for community engagement efforts with faith-based groups, and to implement a national faith-focused CoVPN education program that used anti-racism, anti-homophobic, anti-transphobic, and other principles to ensure that the activities and messages reached broad audiences. Six Faith Ambassadors representing clergy from a variety of faith traditions were identified across the US to support educational activities and speak to the intersection between faith and science, with

a focus on BIPOC communities. Faith Ambassadors worked closely with more than 30 regional faith leaders to engage congregations and faith-based organizations, and to identify additional channels for message dissemination.

Part V: Communications and community influencers

Following the launch of the CoVPN website and Registry in July 2020, an extensive marketing and communications campaign launched in September 2020 to address COVID-19 vaccine trial concerns. The campaign focused on adults over 50 years old, Latino/a/Hispanic, and Black/African American communities. It was developed using audience insights and testing gained through in-depth one-on-one interviews and surveys conducted in English and Spanish with members of the priority populations. The campaign employed a robust media mix including TV, connected TV, radio, internet audio, digital platforms and social media, as well as partnerships and sponsorships with trusted organizations such as the American Association of Retired People, BlackDoctor.org and celebrity personalities. Under the umbrella theme, “Help End the Uncertainty,” the campaign consisted of hundreds of content pieces in Spanish and English – broadcast and digital ads, sponsored content, videos, quizzes, interviews, testimonials – and advertising spots that combined user-generated testimonials with a Harrison Ford voice-over. The campaign achieved over 500 million gross impressions, resulting in over 5 million website visits.

Public requests for greater transparency about the clinical trials, calls for explanations of the vaccine science in lay language, and the need for the voices of leading scientists to speak more directly to communities drove the development of content for the communications platform. To respond to these requests, the CoVPN established a blog, “COVID-19 Vaccine Matters,” initially housed on the Johns Hopkins Coronavirus Resource website (https://coronavirus.jhu.edu), and later hosted jointly with the University of Washington. The blog was launched in November 2020 and has steadily grown in readership to a current 22,000 readers of each blog post (March 2021). The blog provided clear, current, and engaging information on the trials as they progressed, addressing issues of concern and giving stakeholders a “front-row seat” as the science unfolded.

To assess the effectiveness of the community engagement strategy, we analyzed the racial and ethnic demographic data of enrolled participants across the Moderna, AZ, Janssen, and Novavax trials for the US-based CoVPN-affiliated CRSs only. The full data reflecting other independently contracted CRSs are not available for all study sponsors. The CoVPN sites were generally more successful in recruiting BIPOC participants (Figures 3 and 5). Across the trials, enrollment was defined differently depending on the trial sponsor: as participant randomization and/or completion of Study Day 1 with intention of continuing (Moderna, AZ, Janssen), or completion of Study Visit 2 (Novavax).

Results

Across the four clinical trials for which data are available, 47% of participants enrolled at CoVPN sites in the US were BIPOC (Fig 1). This included 2% American Indian/Alaska Native, 15% Black/African American, 0.36% Hawaiian/Pacific Islander and 7% Asian. A total of 5,485 (20%) identified as Hispanic/Latino/a. Across the trials, enrollment of White participants ranged from

44% (Moderna) to 56% (AZ), and the enrollment of BIPOC communities closely mirrored their composition in the larger US population.

Data for the CoVPN sites are available for the Moderna trial, the first trial to begin and complete enrollment. These data show a relatively lower rate of enrollment across all BIPOC groups in the first week of enrollment (Fig 2). Within two weeks, however, White enrollment began to quickly outpace enrollment of BIPOC participants, and this continued throughout the accrual period. Although BIPOC enrollment increased over time, it never approached the rate of White enrollment. This reality required actions to be taken to ensure that there were enough allocated enrollment slots remaining to be filled by BIPOC individuals. As a result, all CRSs were instructed to first slow (September 11, 2020), and then halt (September 30, 2020) White enrollment. These actions allowed the remaining enrollment slots to be filled by BIPOC individuals, thus ensuring that eventual safety and efficacy data would be relevant to the U.S. populations where it was needed most.

In the AZ (Fig 3) and Janssen (Fig 4) trials, White enrollment again outpaced that of BIPOC participants. Enrollment across all BIPOC groups was low during the first month of enrollment and after a study safety pause in both trials, enrollment was slow to resume for two more weeks, followed by a sharp uptick in enrollment. As with Moderna, BIPOC enrollment began slowly but was steady, always outpaced by White enrollment. Focused and intentional efforts to enroll BIPOC individuals were accelerated in the final weeks of the trials to ensure that remaining enrollment slots were filled by BIPOC individuals.

The Novavax trial, which opened several months after the other trials, benefitted considerably from ongoing community engagement efforts, and greater and more diverse volunteer registry records. This was particularly true of efforts to partner with tribal leaders to address data sovereignty and ownership, resulting in increased participation among Indigenous peoples. As a result, although enrollment of BIPOC individuals was always outpaced by that of White individuals, it was constant throughout the Novavax accrual period (Fig 5).

Discussion

Intentional and robust community engagement efforts are critical to ensuring the equitable inclusion of BIPOC communities in vaccine clinical trials. These data illustrate that with ample resources and community engagement expertise, the equitable enrollment of BIPOC individuals can be achieved. What is also clear, however, is that even with robust fiscal resources and a longstanding collaborative and collective effort, the enrollment of White persons outpaces that of BIPOC communities. Without established recruitment goals that reflect the slower yet steady pace of BIPOC enrollment, the allocated enrollment slots were quickly filled, effectively blocking BIPOC persons' opportunities for participation. Rather than directing sites to slow or halt White enrollment, which presents its own operational challenges, future vaccine clinical trial efforts must include clear established goals for BIPOC enrollment from the outset of study accrual, reserving space in the trial to ensure equitable inclusion. The establishment of recruitment goals has achieved remarkable success in recruiting BIPOC and transgender participants in HIV Prevention Trials Network (HPTN) 083, an HIV prevention trial that set specific and measurable goals for the enrollment of transgender women and Black cisgender men who have sex with men[23].

Another approach to ensuring equity is the development of population-specific trials. HPTN 091 will be the first HPTN study designed specifically and exclusively for transgender women. AIDS Clinical Trials Group (ACTG) A5366 is the first HIV cure-related study designed specifically and exclusively for cisgender women. The ACTG is also currently developing a study exploring the effect of gender-affirming hormones on the HIV reservoir in transgender women living with HIV. These population-specific approaches ensure the inclusion of under-represented populations in research that could benefit them.

As seen in the Novavax trial, it is clear that prolonged and directed engagement with priority communities can yield equitable inclusion. When this is the reality across clinical research, the establishment of recruitment goals and population-specific trials may no longer be necessary, as equitable inclusion will be the norm and not the exception. To this end, the recent publication by the U.S. Food and Drug Administration of guidelines for the pharmaceutical industry to emphasize diversity of enrollment in clinical trials is a welcome step[24].

Acknowledgments

This research was supported by the National Institutes of Health. We thank the over 130,000 individuals who volunteered in these trials. We are grateful to Jasmine Hwang and Rohit Banerjee from SCHARP for their assistance in the creation of the datasets and Mindy Miner for her editing assistance. We thank the CoVPN Expert Panelists and Community Working Group members for their unwavering commitment to their communities and their guidance and direction. We also thank the CoVPN Community Engagement team for its collaborative efforts and commitment to equity and inclusion.

Disclaimer

The views expressed in this article are those of the authors and should not be construed to represent the official positions of their affiliated institutions, the US Army, the Department of Defense, or the National Institutes of Health. Dr. Chris Beyrer received support from the CoVPN for his community engagement work.

References

1. Agency for Healthcare Research and Quality. 2019 National Healthcare Quality and Disparities Report. AHRQ. https://www.ahrq.gov/research/findings/nhqrdr/nhqdr18/index.html. Published 2020. Accessed March 25, 2021.
2. National Center for Health Statistics. *Health, United States, 2015: With Special Feature on Racial and Ethnic Health Disparities.* Hyattsville (MD): National Center for Health Statistics; May 2016.
3. Poteat T, Millet G, Nelson L, Beyrer C. Understanding COVID-19 risks and vulnerabilities among black communities in America: the lethal force of syndemics. *Ann Epidemiol.* 2020;47:1-3.
4. UNAIDS (Joint United Nations Programme on HIV/AIDS), AVAC (AIDS Vaccine Advocacy Coalition). Good participatory practice: guidelines for biomedical HIV prevention trials. UNAIDS. https://www.unaids.org/sites/default/files/media_asset/JC1853_GPP_Guidelines_2011_en_0.pdf. Published 2011. Accessed March 25, 2021.
5. Bronfenbrenner U. *The Ecology of Human Development: Experiments by Nature and Design.* Harvard University Press; 1979.
6. Prochaska J, DiClemente C. Stages and processes of self-change of smoking: toward an integrative model of change. *J Consult Clin Psychol.* 1983;51(3):390-395.
7. Rivet Amico K. A situated-Information Motivation Behavioral Skills Model of Care Initiation and Maintenance (sIMB-CIM): an IMB model based approach to understanding and intervening in engagement in care for chronic medical conditions. *J Health Psychol.* 2011;16(7):1071-1081.
8. Andrasik M, Chandler C, Powell B, et al. Bridging the Divide: HIV Prevention Research and Black Men Who Have Sex With Men. *Am J Public Health.* 2014;104(4):708-714.
9. Andrasik M, Grove D, Broder G, Scott H, Allen M, Karuna S. A descriptive analysis of transgender participants in phase 1-2a trials of the HIV Vaccine Trials Network (HVTN) in the United States and Peru. *Vaccine.* 2019;37(29):3911-3917.
10. Andrasik M, Yoon R, Mooney J, et al. Exploring barriers and facilitators to participation of male-to-female transgender persons in preventive HIV vaccine clinical trials. *Prev Sci.* 2014;15(3):268-276.
11. Dietrich J, Lazarus E, Andrasik M, et al. Mobile Phone Questionnaires for Sexual Risk Data Collection Among Young Women in Soweto, South Africa. *AIDS Behav.* 2018;22(7):2312-2321.
12. Andrasik M, Sesay F, Isaacs A, Oseso L, Allen M. Social Impacts among Participants in HIV Vaccine Trial Network (HVTN) Preventive HIV Vaccine Trials. *J Acquir Immune Defic Syndr.* 2020;84(5):488-496.
13. Broder G, Lucas J, Davis J, et al. Standardized metrics can reveal region-specific opportunities in community engagement to aid recruitment in HIV prevention trials. *PLoS One.* 2020;15(9):e0239276.
14. Thiam-Diouf A, Metch B, Sharpe C, Mulugeta R, Andrasik M. Substance use patterns of HVTN phase I clinical trial participants: Enrollment, risk reduction counseling and retention. *Vaccine.* 2018;36(9):1235-1242.
15. Huamani K, Metch B, Broder G, Andrasik M. A Demographic Analysis of Racial/Ethnic Minority Enrollment Into HVTN Preventive Early Phase HIV Vaccine Clinical Trials Conducted in the United States, 2002-2016. *Public Health Rep.* 2019;134(1):72-80.
16. Djomand G, Katzman J, di Tomasso D, et al. Enrollment of racial/ethnic minorities in NIAID-funded networks of HIV vaccine trials in the United States, 1988 to 2002. *Public Health Rep.* 2005;120(5):543-548.

17. Flores L, Frontera W, Andrasik M, et al. Assessment of Inclusion of Racial/Ethnic Minority, Female, and Older Individuals in Vaccine Clinical Trials. *JAMA Network Open* 2021;4(2):e2037640-e2037640.
18. van Dorn A, Cooney R, Sabin M. COVID-19 exacerbating inequalities in the US. *Lancet.* 2020;395(10232):1243-1244.
19. COVID Tracking Project, Center for Antiracist Research. COVID-19 is affecting Black, Indigenous, Latinx, and other people of color the most. The COVID Tracking Project at *The Atlantic* https://covidtracking.com/race. Published 2020. Updated June 2, 2020. Accessed March 25, 2021.
20. Wallerstein N, Duran B, Oetzel JG, Minkler M. *Community-Based Participatory Research for Health: Advancing Social and Health Equity.* 3rd ed: John Wiley & Sons; 2017.
21. Global Indigenous Data Alliance. CARE Principles for Indigenous Data Governance. GIDA. https://www.gida-global.org/care. Published 2019. Accessed March 25, 2021.
22. Thakur N, Lovinsky-Desir S, Bime C, Wisnivesky JP, Celedón JC. The Structural and Social Determinants of the Racial/Ethnic Disparities in the U.S. COVID-19 Pandemic. What's Our Role? *Am J Respir Crit Care Med.* 2020;202(7):943-949.
23. Grinsztejn G, Donnell D, Clement M, et al. *Efficacy of Pre-exposure Prophylaxis (PrEP) containing Long-acting injectable cabotegravir (CAB-LA) is maintained across regions and key populations.* Paper presented at the International AIDS Society Conference; July 9, 2020; San Francisco, CA.
24. U.S. Department of Health and Human Services, Food and Drug Administration, Center for Drug Evaluation and Research (CDER), Center for Biologics Evaluation and Research (CBER). Enhancing the Diversity of Clinical Trial Populations — Eligibility Criteria, Enrollment Practices, and Trial Designs Guidance for Industry. U.S. Food & Drug Administration. https://www.fda.gov/regulatory-information/search-fda-guidance-documents/enhancing-diversity-clinical-trial-populations-eligibility-criteria-enrollment-practices-and-trial. Published 2020. Updated November 13, 2020. Accessed March 25, 2021.

Figure 1 Click here to access/download;Figure;Andrasik_Figure 1.docx

Figure 1: US Diverse Enrollment Across the NIH-funded Moderna, AstraZeneca, Janssen, and Novavax trials

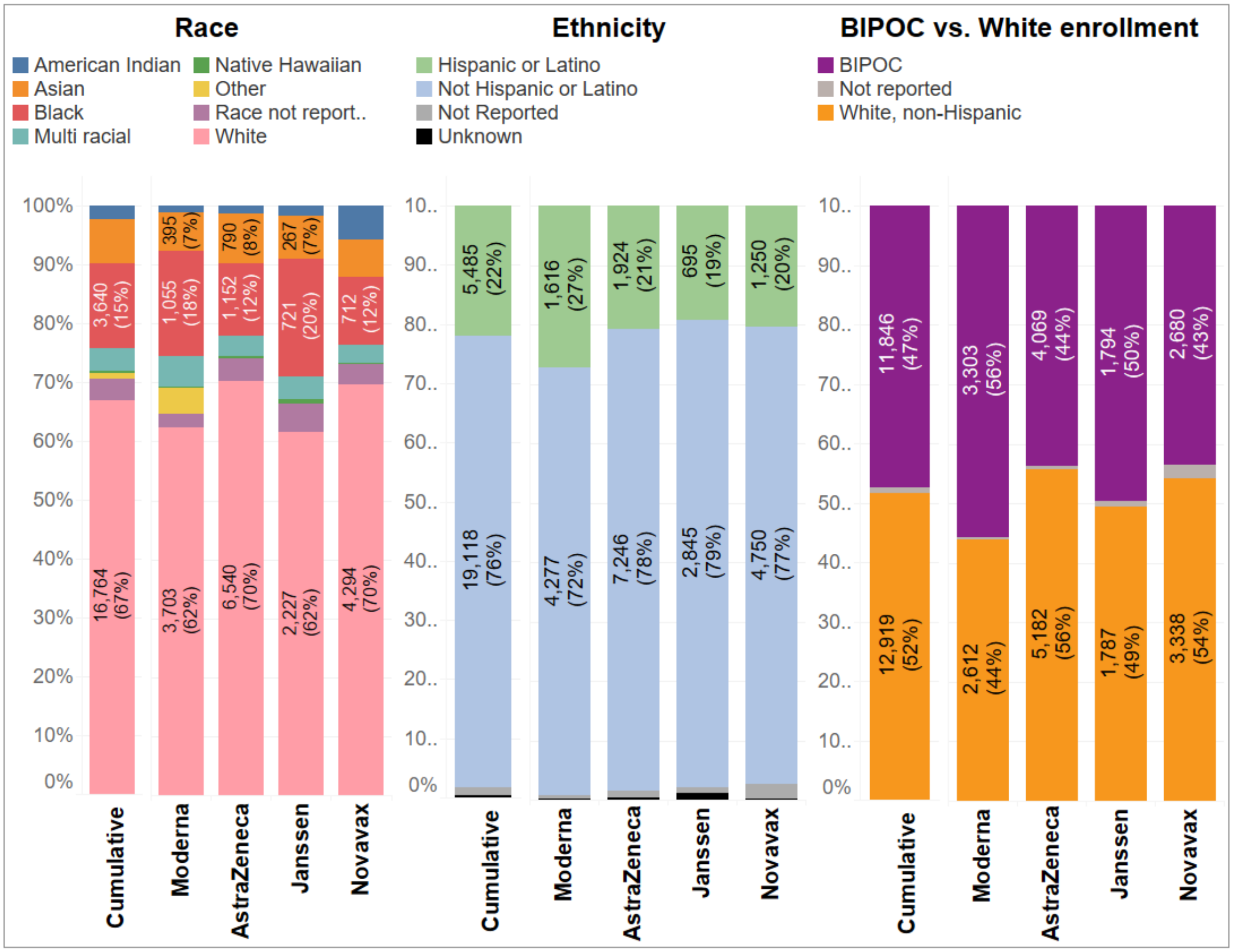

Figure 2

Click here to access/download;Figure;Andrasik_Figure 2.docx

Figure 2: Moderna (Cove Study) Enrollment:

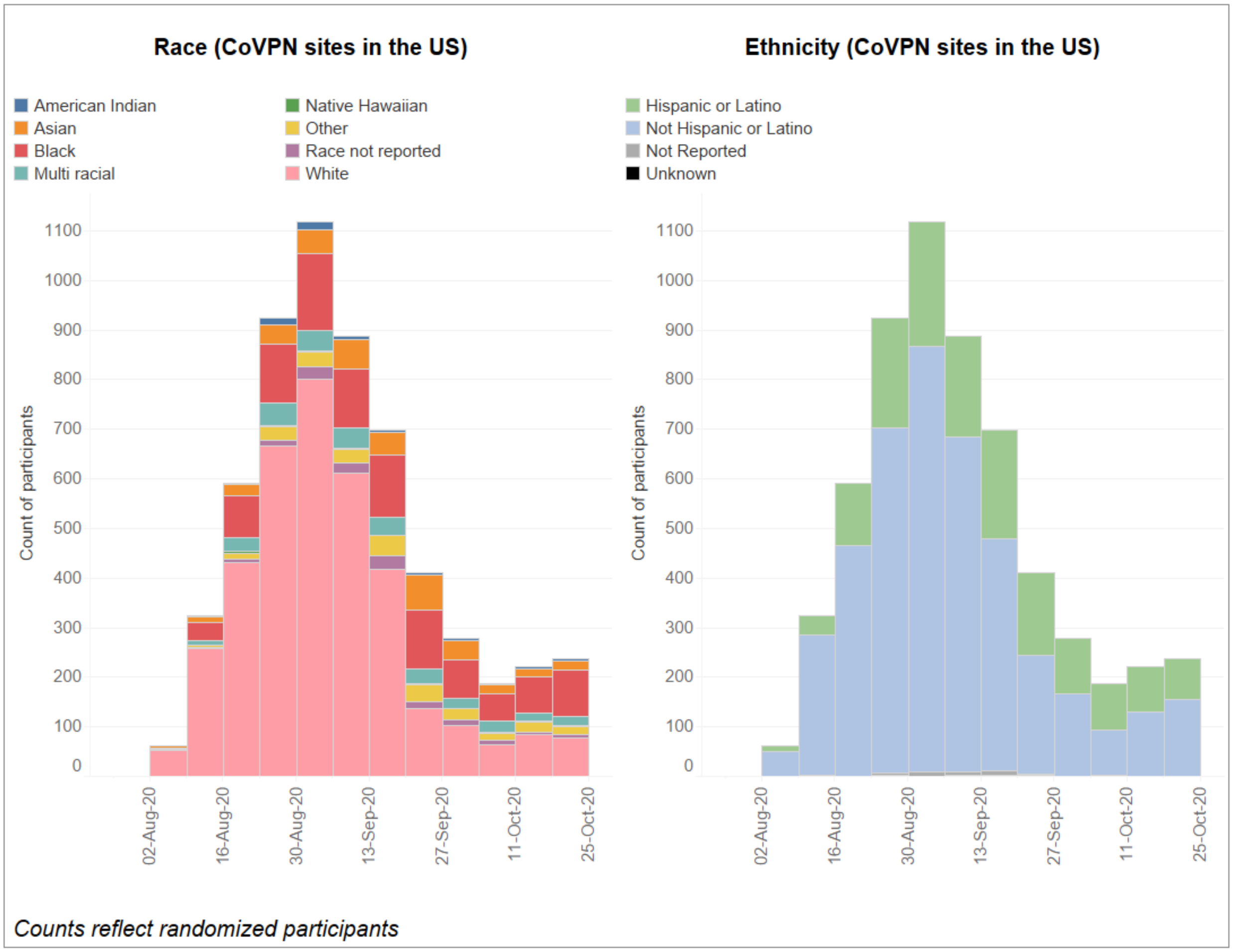

Click here to access/download;Figure;Andrasik_Figure 3.docx

Figure 3: AstraZeneca Study Enrollment

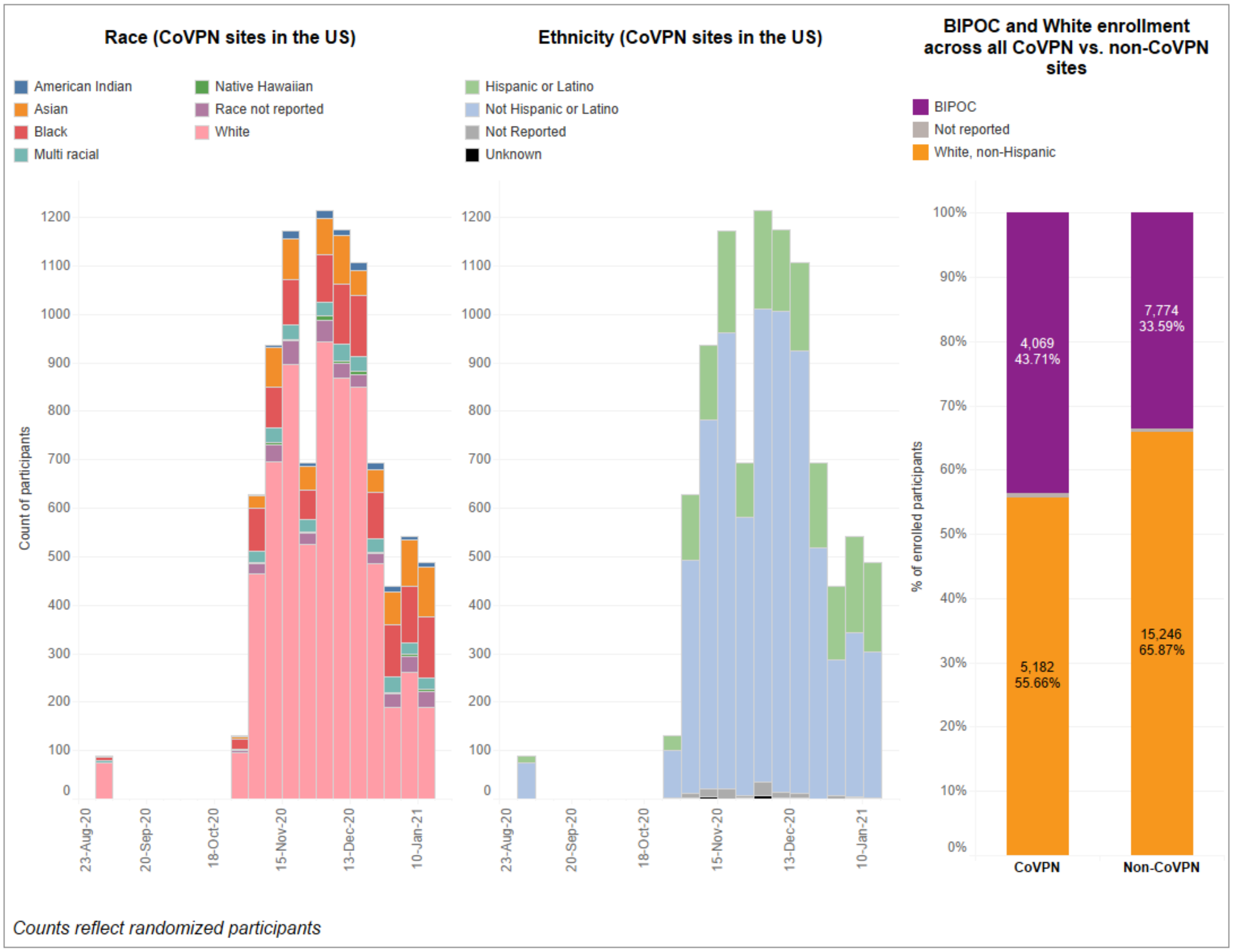

 Click here to access/download;Figure;Andrasik_Figure 4.docx

Figure 4: Janssen (Ensemble Study) Enrollment

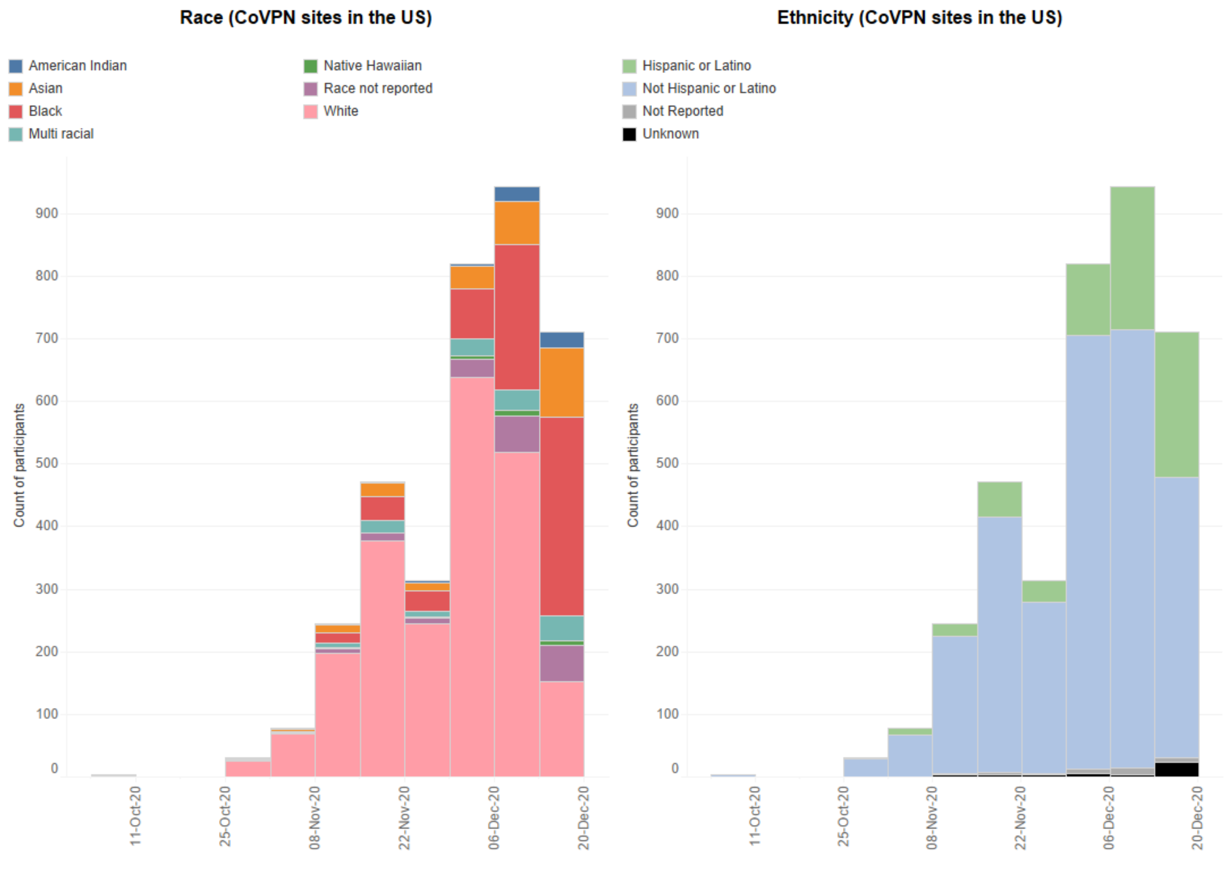

Counts reflect participants who completed Study Day 1 with intention of continuing

Figure 5: Novavax Study Enrollment

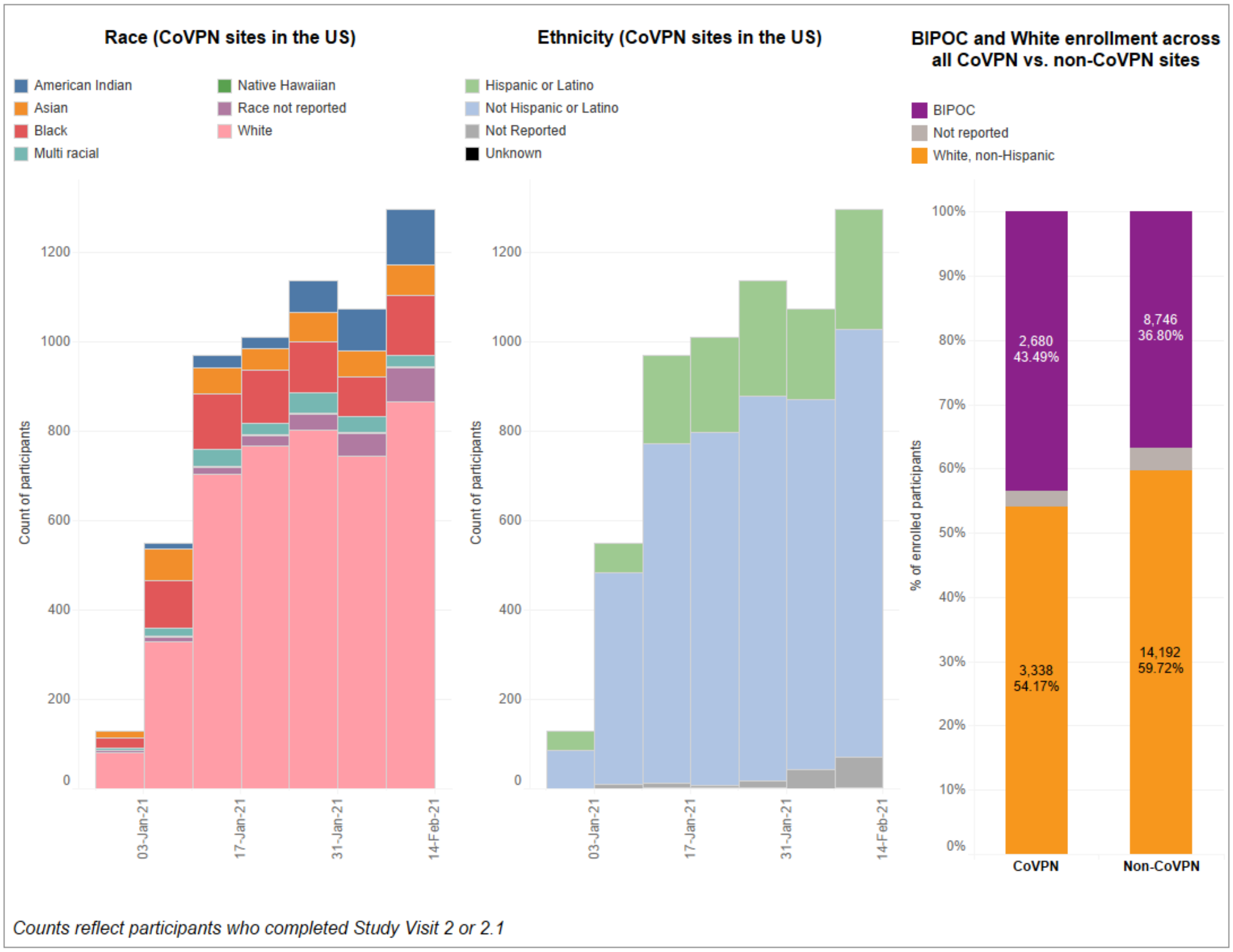

Supplement: Attachment — Submitted filename: PONE-D-21-14138_reviewer_NTH.pdf [file pone.0258858.s001.pdf]
