# Supplementary material for: Increasing Black, Indigenous and People of Color participation in clinical trials through community engagement and recruitment goal establishment
Source: PLoS One. 2021 Oct 19;16(10):e0258858. doi: 10.1371/journal.pone.0258858 (PMC8525736; doi:10.1371/journal.pone.0258858)
Supplement: Attachment — Submitted filename: Andrasik_ResponsetoReviewers_PLOSONE_Jul2021_Final.pdf [file pone.0258858.s002.pdf]

HIV VACCINE
TRIALS NETWORK

FRED HUTCH

MICHELE P ANDRASIK, PH.D.
Director, Social & Behavioral Sciences and Community Engagement, HIV Vaccine Trials Network
Senior Staff Scientist, Vaccine and Infectious Disease Division, Fred Hutch

July 20, 2021

Peter A. Newman Ph.D.
Academic Editor
PLOS One

PLOS ONE Manuscript No.: PONE-D-21-14138

Title: Increasing Black, Indigenous and People of Color Participation in Clinical Trials Through Community Engagement and Recruitment Goal Establishment

Thank you for your thorough review of our manuscript. We appreciate the thoughtful feedback and have incorporated the suggested revisions to improve the manuscript. We have addressed each of the reviewers' concerns in a point-by-point fashion below and have provided line numbers (in the manuscript with tracked changes) for each revision. Additionally, we conducted a thorough review and enlisted the services of a technical editor, to ensure that there are no grammatical or spelling errors. In these reviews, we have made additional revisions to the manuscript and minor changes to the abstract to improve readability.

REVIEWER 1:

The authors mention the "Good Participatory Practice" guidelines; could these be described briefly for a general readership that may not be familiar with these HIV specific guidelines?

> On page 3, lines 97-103 we have inserted: "In 2011, the Joint United Nations Programme on HIV/AIDS (UNAIDS) and AVAC developed the GPP guidelines to standardize practices globally for stakeholder engagement in biomedical HIV prevention trials. "The GPP guidelines set global standard practices for stakeholder engagement. When applied during the entire lifecycle of a biomedical trial, they enhance both the quality and outcomes of research."[4] The GPP framework has recently been adapted by the World Health Organization to emerging pathogens.[9]"

Within "Part 1" (particularly the first paragraph) – it would be helpful to see more specificity in terms of what the CBPR efforts looked like in terms of community providing feedback (vs. just the outcomes of the feedback that they provided). This might help other researchers to better emulate the authors' efforts.

> We have combined parts I and II, as many of the activities that were included in the CBPR efforts were captured in part II. As the reviewer states below, what is described in

both parts is our use of CBPR approaches.

Pg. 4, line 145, "the registry has over 600,000 diverse individuals…" Would be helpful to define what they mean by "diverse" (e.g., race/ethnicity? Sexual orientation? Age?)

We removed "diverse" in describing the registry. The data have not been appropriately cleaned to report on demographics or clinical trials representation and will be included in a publication later in the year.

"Part II – Involving Communities" – could the authors clarify how this is different than Part I (CBPR), which also is focused on community involvement? For example, Part II describes building relationships with groups through Historically Black Medical Colleges and engaging tribal/indigenous communities, which sounds closely aligned with a CBPR approach.

We agree with the reviewer's point and have combined Parts I and II.

"Part III – stakeholder engagement" – it may help readers to replicate the authors' efforts to describe what interpersonal skills/behaviors they used in order to demonstrate "humility and authenticity" to participants.

On page 6, lines 228-232, we have added the following to provide guidance: "This involves knowing and understanding community context and needs, actively listening to fears and concerns of community members, being truthful and transparent at all times, ensuring that all information is provided in plain language, and making resources available from trusted sources to help community members make informed decisions."

"Part V" mentions in-depth one-on-one interviews and surveys to shape the marketing and communications campaign. If the findings from these interviews and surveys have been published somewhere (even if not in an academic journal), it would be great to reference them.

We have not published the data from the audience research, but plan to do so in the future.

The same paragraph mentioned reaching over 500 million gross impressions and 5 million website visits – do the authors have any data on who (e.g., demographically) was reached?

We added, this sentence on page 7, lines 282-283: "The advertising buy focused on Black and Latino/a adults, aged 45 and older, who live in the US and speak either English or Spanish.

Relatedly, the authors mention developing a blog to reach lay audiences regarding the vaccine. Do they have any data on who the readership (e.g., demographically) of the blog was?

We are not collecting demography of the readership. We are running at about 20,000 readers per blog post.

It would be helpful to clarify what the ideal enrollment would have been for these trials in terms of enrolling BIPOC participants. For example, do the authors believe that ideally, representation of different groups should reflect the US population? The proportion of the population affected by COVID-19? This comes up in the discussion too, when the authors refer to "the equitable enrollment of BIPOC individuals." How do the authors define "equitable

enrollment" (e.g., equitable in terms of representation reflecting the impact of COVID by demographic group?)?

On page 9, lines 362-369, we have provided language explaining our view of equitable enrollment: "Equitable inclusion requires representation of BIPOC participants reflecting the composition of the US population in situations where disease impact is equally distributed across communities. However, an approach that enrolls BIPOC participants at a rate that reflects the disproportionate impact of the disease on specific populations would represent an optimized framework for clinical trial enrollment objectives. If the intended use of a biomedical intervention is more impactful in certain communities, then the clinical trial enrollment should be informed by the eventual intended use of such biomedical interventions."

The authors provide important recommendations to help with planning future clinical trials, such as setting "clear established goals for BIPOC enrollment…" Do the authors have recommendations, based on their experience, on what these goals should be (or guidelines on how to determine what these goals should be) in order to achieve equitable representation?

We have inserted the following paragraph with specific recommendations on page 9, lines 391-397: "When conducting clinical trials, research teams can utilize the framework proposed by Bolen et al (2006) to select consistent a priori recruitment goals for underrepresented groups based on the research question and study location.[26] The Division of AIDS (DAIDS)-funded Office of HIV/AIDS Network Coordination (HANC) Legacy Project recently developed the Representative Studies Rubric tool to ensure representation of priority populations in the development of research protocols[27], and advocacy efforts are underway to ensure the adoption of this rubric for DAIDS-funded research.

Minor: Would be great to reduce use of lesser known acronyms (e.g., CRS for clinical research sites)

We have replaced all instances of "CRS" with "clinical research site."

REVIEWER #2:

Some proofreading is needed. I made several suggestions in the attached, but a careful review would help.

Thank you for your suggested revisions. We have addressed all of your suggested revisions and have carefully reviewed the manuscript and enlisted the assistance of a technical editor to ensure there are no grammatical errors.

The background would benefit from some data on underrepresentation of BIPOC populations in clinical trials.

We agree and on page 2, lines 67-74, we have inserted: "A study examining US-based vaccine trials registered on ClinicalTrials.gov from July 1, 2011 through June 30, 2020 found that, among adult studies, Black, African American, American Indian, Alaska Native and Hispanic/Latino/a individuals were underrepresented compared

with US census data.[4] In pediatric trials, Black, African American and Hispanic/Latino/a participants were underrepresented. In addition, among the pediatric trials reporting race and ethnicity, almost half did not report American Indian or Alaska Native participants and over 60% did not include Hawaiian or Pacific Islander participants. [4]

A brief discussion of how data on race and ethnicity were collected in the trials and presented in the paper would be of help. It appears they were collected separately and per OMB and that people were able to select multiple races, but audiences/readers are consistently confused by these data, particularly when race and ethnicity are presented separately as is done here.

We have provided the following sentence in the methods section on page 7, lines 300-303: "Racial and ethnic category data were collected based on the established NIH-required racial and ethnic categories and definitions.[24] Race focuses on physical characteristics, particularly skin color, whereas ethnicity attempts to capture a group's cultural identity.

Discuss the fact that enrollment of BIPOC was substantially higher in CoVPN than non-CoVPN sites. Was the difference stat significant?

No analysis was done to determine statistical significance of the difference in pace in the enrollment of white versus BIPOC individuals between CoVPN and non-COVPN sites. We have limited access to non-CoVPN site data across the trials and as such are unable to conduct these analyses. The CoVPN sites have a longstanding history of conducting clinical trials for HIV, another pandemic where racial disparities are pronounced. The CoVPN site staff are accustomed to making this effort and have built the community relationships over many years that support them in being successful.

Sentence on higher enrollment of BIPOC in CoVPN sites belongs in the results.

We have moved this sentence to the results section. It is now on page 8, lines 351-353.

I provided a few suggestions in the tracked-changes document to increase clarity of how findings are described.

Thank you. We have accepted all of the proposed revisions.

May want to point out that Week 1 enrollment was low across all trials and groups.

We have added this sentence in the results on page 7, lines 311-312: "Across all trials and racial/ethnic groups, enrollment at week 1 was low."

Addressing data sovereignty and ownership is mentioned in 2-3 places in the paper, would be helpful to specify how it was addressed or at least provide an example from one trial.

We have added the following sentence to page 5, lines 182-188: "The NIH Tribal Health Research Office, the CoVPN Regulatory Affairs unit, and the pharmaceutical sponsors worked with Tribal nations to develop contracts that outlined tribal data, material, biospecimen sharing and ownership agreements. These contracts took time to negotiate and were not fully executed until the Moderna and Janssen trials

were almost fully enrolled. The noticeable increase in AI/AN participants in the Novavax trial is likely due to these contracts being in place earlier during study accrual.”

Well written and argued. I only struggled with this sentence because I think it is an overstatement: "When this is the reality across clinical research, the establishment of recruitment goals and population-specific trials may no longer be necessary, as equitable inclusion will be the norm and not the exception." Given that disparities in healthcare treatment and access would persist even in this case, I suggest the authors instead point out that ongoing commitment to these standards and partnerships will decrease the cost involved in community engagement for any one study.

We have addressed this. Lines 399-402 on page 9 now reads: “Ongoing commitment to these standards and partnerships will help communities view researchers and research institutions as trustworthy, and build and strengthen rapport between communities, researchers and research institutions.”

Figures:
I am unclear where Alaska Natives and Pacific Islanders are in these figures, if at all.

We collected all race and ethnicity data according to NIH-required guidelines. “American Indian or Alaska Native” are in the same category. Similarly, “Native Hawaiian or Other Pacific Islander” are in the same category. Both categories were renamed in the revised figures.

Why is it that there are data provided for people with unknown ethnicity, but not unknown race, especially given that Latinos often select unknown or do not specify race?

Race ‘unknown’ was grouped together with ‘Race not reported’. We have separated them out in the revised figures.

On the left panel of the 1st & 5th figure, orange is for Asians. However, on the right panel orange is for non-Hisp/Whites. I suggest using a different color for one.

The colors have been standardized in both figures. Asian representation is now illustrated in blue.

Sincerely,

Michele Andrasik

Michele Peake Andrasik, PhD
1100 Fairview Avenue North
E3-300
PO Box 19024
UW Box#: 358080
Seattle, WA 98109
mandrasik@fhcrc.org
206-667-2074
